# Extracorporeal cardiopulmonary resuscitation versus standard treatment for refractory out-of-hospital cardiac arrest: a Bayesian meta-analysis

**DOI:** 10.1186/s13054-024-05008-9

**Published:** 2024-07-03

**Authors:** Samuel Heuts, Johannes F. H. Ubben, Michal J. Kawczynski, Andrea Gabrio, Martje M. Suverein, Thijs S. R. Delnoij, Petra Kavalkova, Daniel Rob, Arnošt Komárek, Iwan C. C. van der Horst, Jos G. Maessen, Demetris Yannopoulos, Jan Bělohlávek, Roberto Lorusso, Marcel C. G. van de Poll

**Affiliations:** 1 Department of Cardiothoracic Surgery, Maastricht University Medical Center, MUMC+), P. Debyelaan 25, 6229HX Maastricht, The Netherlands; 2 Cardiovascular Research Institute Maastricht (CARIM), University Maastricht, Maastricht, The Netherlands; 3 Department of Anesthesiology and Pain Medicine, Maastricht University Medical Center (MUMC+), Maastricht, The Netherlands; 4 Department of Methodology and Statistics, University Maastricht, Maastricht, The Netherlands; 5 Care and Public Health Research Institute (CAPHRI), University Maastricht, Maastricht, The Netherlands; 6 Department of Intensive Care Medicine, Maastricht University Medical Center (MUMC+), Maastricht, The Netherlands; 7 2nd Department of Medicine-Department of Cardiovascular Medicine, First Medical School, General University Hospital and Charles University in Prague, Prague, Czech Republic; 8 Department of Probability and Mathematical Statistics, Faculty of Mathematics and Statistics, Charles University in Prague, Prague, Czech Republic; 9 Center for Resuscitation Medicine, University of Minnesota Medical School, Minneapolis, MN USA; 10 School of Nutrition and Translational Research in Metabolism, University Maastricht, Maastricht, The Netherlands

**Keywords:** Out-of-hospital cardiac arrest, Extracorporeal cardiopulmonary resuscitation, Conventional cardiopulmonary resuscitation, Neurologically favorable survival, Randomized controlled trials, Bayesian statistical inference

## Abstract

**Background:**

The outcomes of several randomized trials on extracorporeal cardiopulmonary resuscitation (ECPR) in patients with refractory out-of-hospital cardiac arrest were examined using frequentist methods, resulting in a dichotomous interpretation of results based on p-values rather than in the probability of clinically relevant treatment effects. To determine such a probability of a clinically relevant ECPR-based treatment effect on neurological outcomes, the authors of these trials performed a Bayesian meta-analysis of the totality of randomized ECPR evidence.

**Methods:**

A systematic search was applied to three electronic databases. Randomized trials that compared ECPR-based treatment with conventional CPR for refractory out-of-hospital cardiac arrest were included. The study was preregistered in INPLASY (INPLASY2023120060). The primary Bayesian hierarchical meta-analysis estimated the difference in 6-month neurologically favorable survival in patients with all rhythms, and a secondary analysis assessed this difference in patients with shockable rhythms (Bayesian hierarchical random-effects model). Primary Bayesian analyses were performed under vague priors. Outcomes were formulated as estimated median relative risks, mean absolute risk differences, and numbers needed to treat with corresponding 95% credible intervals (CrIs). The posterior probabilities of various clinically relevant absolute risk difference thresholds were estimated.

**Results:**

Three randomized trials were included in the analysis (ECPR, n = 209 patients; conventional CPR, n = 211 patients). The estimated median relative risk of ECPR for 6-month neurologically favorable survival was 1.47 (95%CrI 0.73–3.32) with a mean absolute risk difference of 8.7% (− 5.0; 42.7%) in patients with all rhythms, and the median relative risk was 1.54 (95%CrI 0.79–3.71) with a mean absolute risk difference of 10.8% (95%CrI − 4.2; 73.9%) in patients with shockable rhythms. The posterior probabilities of an absolute risk difference > 0% and > 5% were 91.0% and 71.1% in patients with all rhythms and 92.4% and 75.8% in patients with shockable rhythms, respectively.

**Conclusion:**

The current Bayesian meta-analysis found a 71.1% and 75.8% posterior probability of a clinically relevant ECPR-based treatment effect on 6-month neurologically favorable survival in patients with all rhythms and shockable rhythms. These results must be interpreted within the context of the reported credible intervals and varying designs of the randomized trials.

**Registration:**

INPLASY (INPLASY2023120060, December 14th, 2023, https://doi.org/10.37766/inplasy2023.12.0060).

**Supplementary Information:**

The online version contains supplementary material available at 10.1186/s13054-024-05008-9.

## Background

Following encouraging results derived from observational studies [1, 2], several randomized controlled trials (RCTs) comparing extracorporeal cardiopulmonary resuscitation (ECPR) to conventional cardiopulmonary resuscitation (CCPR) have been conducted [3–5]. A patient-level pooled analysis of two of these trials [6]—and several other meta-analyses [7–12]—were published following the publication of these RCTs, focusing on the potential presence of a statistically significant difference in (neurologically favorable) survival. Still, such analyses are heavily dependent on the sample size and event rate, and may be underpowered to detect a clinically relevant treatment effect. To augment the pooled sample size, some of these meta-analyses have also included non-randomized data [11–14]. However, the addition of such studies increases the risk of various forms of bias that are associated with a non-randomized trial design (including treatment selection bias and allocation bias). Moreover, by expanding the sample size, a potentially established statistically significant difference (based on the *p*-value) may no longer be clinically relevant.

All of the previously performed pooled analyses have applied the frequentist statistical framework, which relies on well-known concepts such as null hypothesis significance testing and *p*-values [15]. In this paradigm, the treatment of interest is considered a ‘fixed parameter’ or ‘fixed points estimate’, and the data is considered ‘unknown’. In that light, the *p*-value denotes the probability of observing the trial data—or more extreme—in future experiments, conditional on the assumption that the null hypothesis is true (i.e., analogous to the probability of a test result, given a disease [16, 17]). Still, the frequentist approach presents several cognitive difficulties, such as, the (in)correct interpretation of the *p*-value [15, 17–19]. Indeed, a *p*-value below the threshold of statistical significance (i.e., < 0.05) does not imply that a treatment effect is definitely clinically relevant, while a *p*-value above the threshold of statistical significance (i.e., > 0.05) does not mean a treatment is necessarily clinically ineffective [16]. Some of these difficulties can be addressed by the application of Bayesian statistical inference. In Bayesian statistical inference, the treatment effect (or hypothesis) is considered 'unknown' and has a probability distribution reflecting its uncertainty. Instead, the data is observed, and therefore 'known'. Using this posterior probability distribution, the Bayesian statistical paradigm allows the estimation of the probability of a treatment effect (or hypothesis) in the light of the observed data. Inherently, this inverse probability thinking resembles clinical reasoning (i.e., analogous to the probability of the disease, given the test result). Therefore, Bayesian statistical inference can be used to estimate the probability of clinically relevant treatment effects, particularly in trials evaluating a resource-intensive treatment such as ECPR, which may be subjected to small sample sizes and low statistical power.

Thus, the researchers behind three recent RCTs conducted a joint systematic review and Bayesian meta-analysis to determine whether there is a clinically relevant effect of ECPR on neurologically favorable survival in patients with refractory OHCA.

## Methods

### Protocol registration

This systematic review and Bayesian meta-analysis was prospectively registered on the international platform of registered systematic reviews and meta-analyses (INPLASY) [20, 21] (registration number: INPLASY2023120060; date of registration: December 14th 2023; 10.37766/inplasy2023.12.0060) and adhered to the Preferred Reporting Items for Systematic Reviews and Meta-Analyses 2020 statement [22].

### Search strategy and study inclusion

A systematic search was performed in MEDLINE, PubMed Central, EMBASE, and the Cochrane Library, including abstract presentations during meetings and preprints (see Supplementary Material 1 for the full search strategy).

Randomized studies were included when comparing ECPR-based treatment to CCPR in patients with refractory OHCA and reporting the primary outcome (neurologically favorable survival at six months). Studies were excluded if they applied a non-randomized design or did not report results on the outcome of neurologically favorable survival. The search was performed by two experienced reviewers (SH and MJK).

### Data extraction

Data extraction was performed by two reviewers using a predefined worksheet, which can be found in Supplementary Material 2 (SH and JFHU).

### Risk of bias assessment

Risk of bias was independently assessed using the Risk of Bias 2.0 tool (RoB 2.0) [23] by two reviewers (SH and JFHU). The final judgment ranged from ‘high’ to ‘low’ risk of bias, based on the RoB 2.0 tool.

### Outcomes

The primary outcome of the current study was 6-month neurologically favorable survival, preferably categorized by the Cerebral Performance Category (CPC) [24]. CPC scores of 1 and 2 were deemed neurologically favorable, and CPC of 3–5 were considered neurologically unfavorable. Alternatively, neurological performance could be assessed using the modified Rankin Scale (mRS) [25], in which an mRS of 0–3 indicated neurologically favorable survival. Given the importance of long-term outcomes and the potential of improvement in neurological status during the first months after refractory OHCA [3], the 6-month time point was considered the primary outcome.

Primary analyses were performed in patients with all rhythms (i.e. refractory OHCA of presumed cardiac origin). Secondary analyses were conducted on patients with shockable rhythms.

The pre-specified outcomes were expressed as mean natural logarithmic (log) relative risks (RRs), median RRs, and mean absolute risk differences (ARDs), accompanied by 95% credible intervals (CrIs), assuming a normal distribution for Bayesian analyses. To enhance clinical interpretation, posterior probabilities of various treatment effect sizes and numbers needed to treat (NNT) were also calculated, with credible intervals denoted as proposed by Altman [26].

### Priors

The primary and secondary analyses were performed under vague priors (i.e. a prior assuming no difference, with a normal and wide distribution capturing plausible treatment effects; please see the statistical analysis section for the prior rationale and specification).

### Minimal clinically important differences

As proposed previously in an expert consensus statement, a 5% absolute risk difference in survival was deemed clinically important in patients with refractory OHCA due to ventricular fibrillation [27, 28]. Thus, the posterior probability of the MCID was evaluated in the primary and secondary analyses. However, as the referenced expert consensus statement by Nichol et al. did not specifically address the question of the MCID in ECPR research [27], the posterior probabilities of the 10%, 15%, and 20% absolute risk difference thresholds were also studied. Notably, these ARDs also captured the range of the trial protocols’ predefined expected treatment effects [29, 30]. ARDs were converted to NNTs (or numbers needed to harm [NNH]) to facilitate clinical interpretation.

### Statistical analysis

Categorical variables were pooled using a random-effects model (using an inverse-variance weighting approach [31]). When continuous variables warranted conversion, Wan’s method was applied [32], and the data were pooled using a random-effects model (inverse-variance weighting) as well. The control group’s (CCPR) risk of survival was considered the baseline risk, and the RR of 6-month neurologically favorable survival in the ECPR group was calculated with CCPR as the reference group. The survival risk of the various trial CCPR groups, also defined as the assumed control risk (ACR), was calculated from a random effects model (expressed as a mean % with corresponding 95% confidence intervals [CIs]). This ACR subsequently facilitated further estimations of ARDs using the approach previously proposed by the Cochrane Collaboration [33, 34]. The rationale and methods for calculating these endpoints are outlined in Supplementary Material 3.

Under a vague prior, no difference between groups is assumed, with a wide distribution of possible treatment effects. As such, the mean RR (mean or μ) of this prior was set to 0 on the log RR scale, and the SD (σ) was set to 2 on the log RR scale. The rationale for this prior selection and prior specification can be found in Supplementary Material 4. All Bayesian hierarchical meta-analyses were performed using a random-effects model. In addition, between-study heterogeneity was assessed using the I^2^ metric and τ^2^ [35].

Posterior probabilities were estimated by use of Markov Chain Monte Carlo (MCMC) sampling algorithms using dedicated openly available software (JASP, version 0.17.1 for Mac) [36], and R statistics using the ‘brms’ package (R Statistics, version 4.2.2, Vienna, Austria), by setting four chains and 10,000 saved iterations per chain.

### Publication bias assessment

Publication bias was assessed visually by inspection of the funnel plots and statistically evaluated by Egger’s test, in which a *p*-value of < 0.05 was considered to indicate the presence of statistically significant publication bias (using Meta-Essentials [37]).

## Results

### Study inclusion

**Fig. 1:**
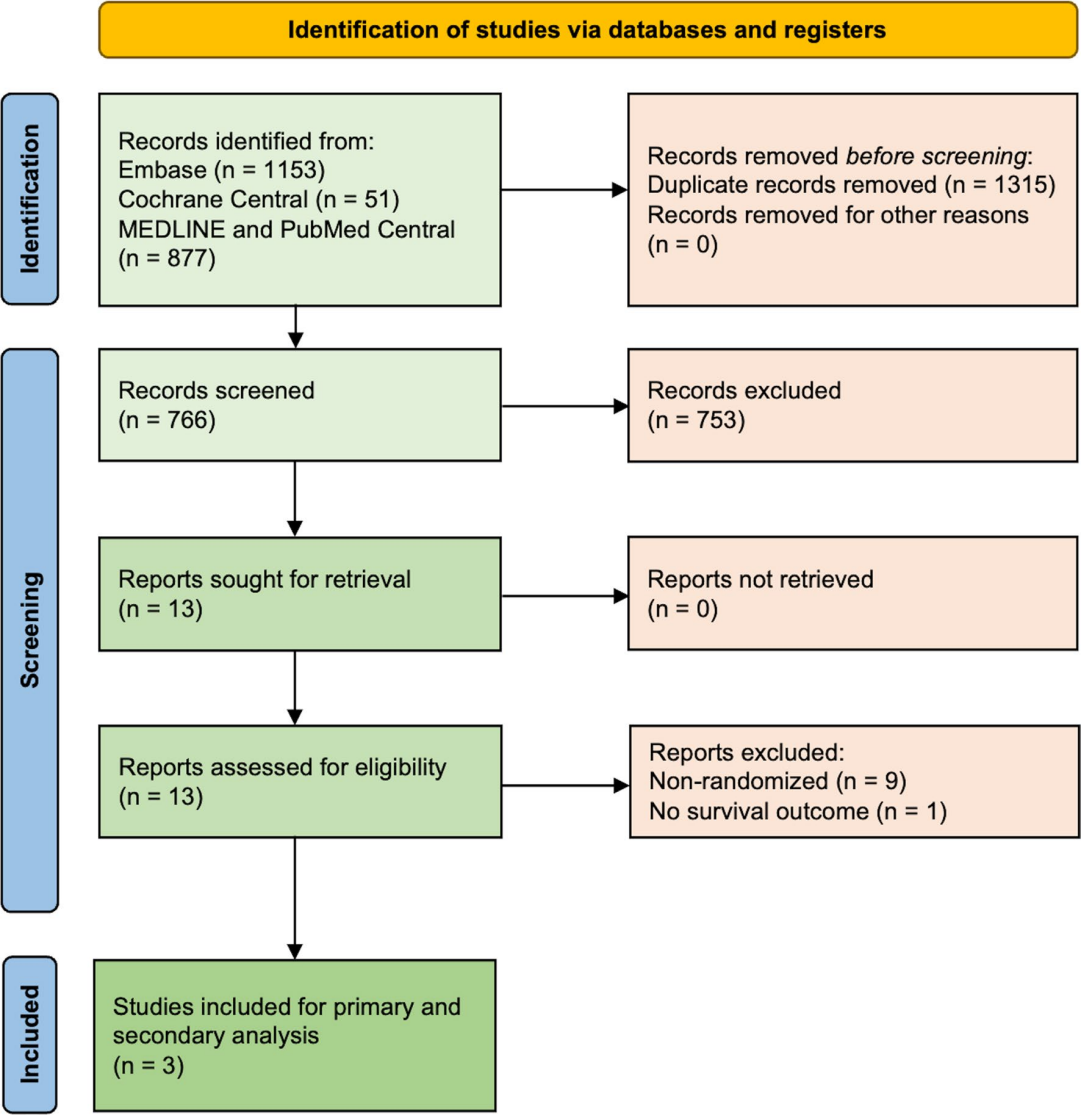
2020 PRISMA flowchart for the study inclusion

Our systematic search yielded 2081 studies across three electronic databases. After removing duplicate studies, 766 studies were screened based on their titles and abstracts. Eventually, 13 studies were retrieved and assessed for eligibility based on full-text assessment. Finally, three randomized trials were included in the primary analysis [3–5] (PRISMA flowchart is presented in Fig. 1).

The ARREST and INCEPTION-trial included patients with shockable refractory OHCA [3, 5], while Prague OHCA included patients with refractory OHCA of presumed cardiac origin (including non-shockable rhythms) [4]. The results of the initial Prague OHCA report were used in the primary analysis, whereas the results of a post-hoc study evaluating the outcomes of patients with shockable rhythms were used in the secondary analysis [38].

### Study and patient characteristics

The three included randomized studies were conducted in the United States [3], Czech Republic [4], and the Netherlands [5] and published between 2020 and 2023. Two trials had a single-center design [3, 4] and one trial had a multi-center design [5].

A total of 420 patients were included in the study (ECPR-based treatment, n = 209 patients; CCPR, n = 211 patients). The mean ages of the patients in the sample were 57 years (SD, 13 years; ECPR group) and 56 years (SD, 12 years; CCPR group). Further studies and patient characteristics are shown in Table 1.

**Table 1:** Study and patient characteristics of the included trials

Parameter	ARREST [4]		Prague OHCA [5]		INCEPTION [6]	
	Extracorporeal	Conventional	Extracorporeal	Conventional	Extracorporeal	Conventional
*Study characteristics*						
Country	USA		Czech Republic		the Netherlands	
Publication year	2020		2022		2023	
Inclusion years	2019–2020		2013–2020		2017–2021	
Design	Single-center		Single-center		Multi-center	
Timing of randomization	In-hospital		Pre-hospital		Pre-hospital	
OHCA type	Refractory and shockable		Refractory of and presumed cardiac origin (shockable and non-shockable)		Refractory and shockable	
Number of patients (n)	15	15	124	132	70	64
*Patient characteristics*						
Age (years)	59 (10)	58 (11)	58 (14)	56 (13)	54 (12)	57 (10)
Sex (female, n, %)	1 (7%)	4 (27%)	22 (18%)	22 (17%)	7 (10%)	7 (11%)
***OHCA cause***						
AMI	–	–	64 (52%)	63 (48%)	51 (73%)	52 (81%)
Other	–	–	60 (48%)	68 (52%)	19 (27%)	12 (19%)
Bystander CPR	13 (87%)	12 (80%)	123 (99%)	129 (98%)	70 (100%)	64 (100%
Shockable rhythm (n, %)	15 (100%)	15 (100%)	72 (58%)	84 (64%)	69 (99%)	63 (98%)
Mechanical CPR (n, %)	15 (100%)	15 (100%)	114 (92%)	104 (79%)	62 (89%)	58 (90%)
Presenting lactate (mmol/L)	11.5 (4.5)	10.7 (3.1)	12.6 (5.1)	10.5 (4.5)	13 (5)	14 (4)
*Neurologically favorable survival*						
30-day CPC 1–2 (n, %)	3 (21%)	0	38 (31%)	24 (18%)	14 (20%)	10 (16%)
6-month CPC 1–2 (n,%)	6 (43%)	0	39 (32%)	29 (22%)	14 (20%)	10 (16%)

Continuous variables are presented as mean and standard deviation

*AMI, acute myocardial infarction; CPC, cerebral performance category; CPR, cardiopulmonary resuscitation; OHCA, out-of-hospital cardiac arrest*

### Risk of bias

The risk of bias assessment showed *some concerns* regarding the presence of the risk of bias, as reported in Supplementary Material 5. These risks mainly arose from bias due to the randomization process or bias due to deviations from the intended intervention.

### Assumed control risk for the CCPR group

For the primary analysis, the ACR was calculated in patients with all rhythms (OHCA of presumed cardiac origin), which yielded a CCPR risk of 6-month neurologically favorable survival of 18.4% (95% CI 11.8–27.7%, I^2^ = 33%, p = 0.228, τ^2^ = 0.077). In the secondary analysis (shockable OHCA), the calculated ACR was 19.9% (95% CI 8.3–40.7%, I^2^ = 76%, *p* = 0.015, τ^2^ = 0.513) in the CCPR group. The calculated ACR facilitated further estimation of the absolute risk differences between the groups.

### Primary analysis: all rhythms

**Fig. 2:**
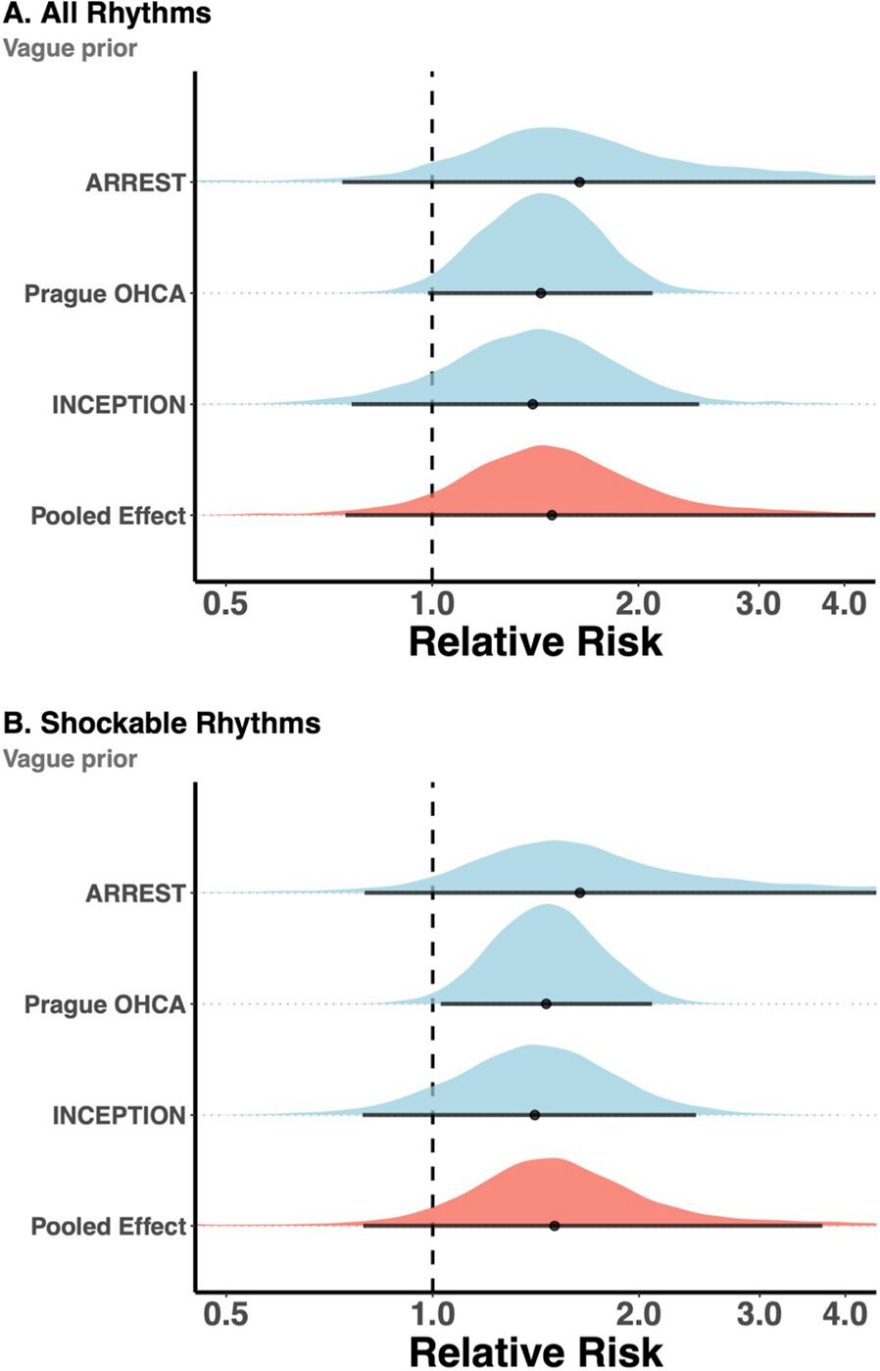
Primary (**A**) and secondary (**B**) Bayesian meta-analyses of primary outcomes under a vague prior. The black horizontal line denotes 95% credible interval

The primary analysis for 6-month neurologically favorable survival in all patients under a vague prior produced a mean log RR of 0.40 (95%CrI − 0.27;1.20) and translated median RR of 1.47 (95%CrI 0.76–3.32, Table 2, Fig. 2A). This resulted in a mean ARD of 8.7% (95%CrI − 5.0; 42.7%), in favor of ECPR-based treatment (NNT 11, 95%CrI 20 ∞ 2, denoted according to Altman [26]). The posterior probability of an ARD of > 0% was 91.0%.

**Table 2:** Primary and secondary analyses under a vague prior

	Relative risks		Posterior probabilities					ARD and NNT	
	Median RR	95%CrI	> 0% ARD	> 5% ARD	> 10% ARD	> 15% ARD	> 20% ARD	Mean ARD (95%CrI)	Mean NNT (95%CrI)
*Primary analysis under the vague prior (all OHCA of presumed cardiac origin)*									
Vague prior	1.47	0.73—3.32	91.0%	71.1%	43.7%	23.5%	13.4%	8.7% (− 5.0; 42.7%)	11 (20 ∞ 2)
Secondary analysis under the vague prior (only shockable rhythms*)									
Vague prior	1.54	0.79—3.71	92.4%	75.8%	50.4%	29.8%	17.6%	10.8% (− 4.2; 73.9%)	9 (24 ∞ 1)

^*^Prague OHCA data for shockable rhythms were derived from Rob et al.[42]

*ARD, absolute risk difference; CrI, credible interval; NNT, number needed to treat*

**Fig. 3:**
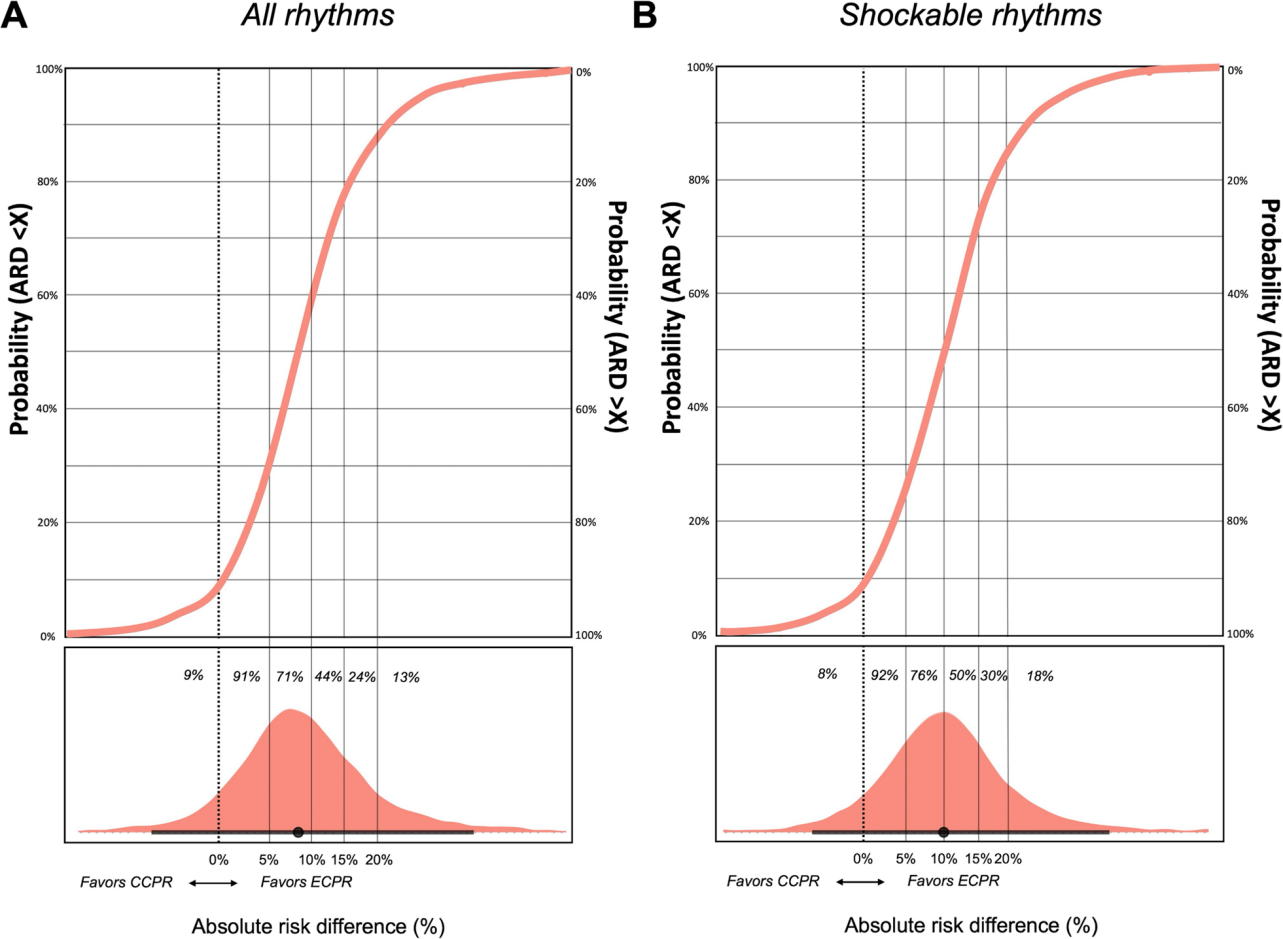
Full posterior probability distribution of the primary (**A**) and secondary (**B**) Bayesian meta-analyses of the primary outcome under a vague prior. The black horizontal line denotes the 95% credible interval. ARD: absolute risk difference, CCPR: conventional cardiopulmonary resuscitation, ECPR: extracorporeal cardiopulmonary resuscitation

The posterior probabilities of an ARD > 5%, > 10%, > 15%, and > 20% were 71.1%, 43.7%, 23.5%, and 13.4%, respectively. The full posterior probability distribution of the absolute risk difference in favor of the ECPR-based treatment is presented in Fig. 3A.

### Secondary analysis under the vague prior (shockable rhythms)

The secondary analysis for 6-month neurologically favorable survival in patients with shockable rhythms under a vague prior estimated a slightly more positive mean log RR of 0.43 (95%CrI -0.23; 1.31) and translated median RR of 1.54 (95%CrI 0.79–3.71) (Table 2, Fig. 2B). This resulted in a mean ARD of 10.8% (95%CrI − 4.2; 73.9%) in favor of ECPR-based treatment (NNT 9, 95%CrI 24 ∞ 1). The posterior probability of an ARD of > 0% was 92.4%. The posterior probabilities of an ARD > 5%, > 10%, > 15%, and > 20% were 75.8%, 50.4%, 29.8%, and 17.6%, respectively (Fig. 3B).

### Publication bias

There was no statistically significant evidence of publication bias (Egger’s test—*p* = 0.239, Supplementary Material 6).

## Discussion

The current Bayesian meta-analysis estimated a 91.0–92.4% posterior probability of any effect of ECPR-based treatment on neurologically favorable survival in patients with refractory OHCA, and a 71.1–75.8% posterior probability of a clinically relevant effect of ECPR-based treatment, in patients with all rhythms and shockable rhythms, respectively. We observed an estimated mean absolute risk difference in 6-month neurologically favorable survival ranging between 8.7% and 10.8% in favor of ECPR-based treatment in these patient groups. Due to the use of the applicable random-effects models and the relatively low number of included patients, credible intervals were wide, illustrating the uncertainty of the estimated effects.

A recent patient-level meta-analysis conducted by the authors of the ARREST and Prague OHCA trials—including 286 patients with refractory OHCA—demonstrated a statistically significant 6-month neurological survival benefit in favor of an ECPR-based treatment (ARD 12.7%, 95% CI 2.6–22.7%, *p* = 0.015) [6]. These results are encouraging and seem to favor the use of ECPR in this patient population, although it must be noted that the two single-center trials were performed at two acknowledged centers with extensive expertise in ECPR. As such, the results of this individual patient data analysis may be less generalizable to ‘real-world’ settings. Simultaneously with ARREST and Prague OHCA, the INCEPTION trial was conducted, a multi-center RCT performed in 10 Dutch centers with established cardiac surgery programs [5]. This trial failed to demonstrate a statistically significant effect of the ECPR-based treatment. Given the multi-center and pragmatic design of the INCEPTION trial, pooling these additional data may enhance the external validity and generalizability of randomized ECPR outcomes, as presented in the current analysis. However, it should be kept in mind that the effectiveness of ECPR-based treatment for OHCA may vary among different systems for the chains of survival.

During the past years, several study-level meta-analyses of these randomized trials have been conducted under a frequentist framework, with or without incorporation of *in-hospital* cardiac arrest patients [13], and with or without incorporation of non-randomized studies to augment the analysis’ power [7–10, 12–14]. When focused on OHCA, the majority of pooled analyses did not observe a statistically significant ECPR treatment effect [7–9, 13]. Still Scquizzato and colleagues reported a statistically significant favorable neurological survival benefit at longest follow-up (odds ratio 1.90, 95% CI 1.16–3.13, p = 0.02). These diverging results seem to be the consequence of heterogeneity in statistical approaches, as the latter study applied a fixed effects model to the pooled analysis [10]. Particularly the use of fixed versus random effects models under the frequentist framework seems disputable since it may not be realistic to assume one true underlying effect size in ECPR research, given the RCTs’ varying study designs [39, 40]. Furthermore, in their commendable updated meta-analysis, Low et al. report a statistically significant benefit of ECPR in refractory OHCA (OR 0.67, 95% CI 0.51–0.88) [12] after the addition of two studies, as compared to their 2023-analysis [13]. Although well-performed, this analysis heavily relies on the incorporation of *non-randomized* data, which subjects the meta-analysis to all biases associated with such designs. Consequently, results may become less reliable. These observations underline the limitations of the frequentist statistical framework, which reduces the interpretation of randomized trials and meta-analyses to a mere a positive or negative result, based on the p-value and the significance level (usually 0.05). In turn, the p-value is determined by the sample size and treatment effect. Indeed, even in case of a small effect, an infinitely large sample size can lead to a statistically significant result (the p-value crossing the 0.05-boundary), while a large treatment effect may not statistically surface in studies or pooled analyses comprising small sample sizes. This is also illustrated by the interpretation of Prague OHCA as a negative trial based on the p-value [4]. Therefore, particularly in a cost- and resource-consuming research-field as refractory OHCA and ECPR, the Bayesian approach may be applicable as it facilitates a more intuitive clinical interpretation of the trials’ results in terms of posterior probabilities and clinically relevant treatment effects.

The Bayesian methodological approach relies on the combination of the ‘prior’ and ‘likelihood’ (i.e., the current data), to obtain the ‘posterior’ [41]. To ensure objectivity, we have opted to perform all our primary analyses under a *vague prior*, which assumes no difference between treatments and considers all possible treatment effects equally likely. As such, the analyses performed in this study are a reflection of the totality of randomized evidence in ECPR literature, and are not influenced by the biases that are associated with non-randomized study designs.

The ARREST trial applied Bayesian inference as the primary statistical approach, declaring the superiority of ECPR if the posterior probability of an ECPR survival benefit exceeded 98.6% during interim analyses [3]. Furthermore, the Prague OHCA and INCEPTION trial reported a post-hoc Bayesian reanalysis of their results [28, 42], following the primary report under the frequentist framework [4, 5]. The Bayesian reanalysis of the Prague OHCA trial demonstrated a posterior probability of *any* effect of ECPR-based treatment (ARD > 0%) of 96.1% for 6-month neurologically favorable survival under a weakly informative prior, but did not study further treatment effect thresholds such as the MCID [42]. The post-hoc analysis of the INCEPTION trial reported a 71.6% posterior probability of *any* effect of ECPR-based treatment and a 42.1% posterior probability of the MCID (ARD > 5%) under a minimally informative prior [28].

Although the credible intervals were wide in the current pooled analysis, our findings seem to support the presence of a clinically relevant effect of ECPR-based treatment in refractory OHCA, with a posterior probability of an ARD of > 5% for 6-month neurologically favorable survival of 71.1% and 75.8% in patients with all rhythms and shockable rhythms, respectively. The MCID of an ARD > 5% was based on a published expert consensus statement by Nichol et al., who evaluated experts’ interpretation of a clinically relevant treatment effect for any intervention applied to patients with refractory shockable OHCA regarding a good neurological outcome at discharge [27]. However, there was high variability in the judgment of this threshold, and this survey was not focused on ECPR, an exceptionally cost- and resource-consuming intervention. Consequently, we also estimated the posterior probabilities of various more extreme treatment effect size ranges, including > 10%, 15%, and > 20% ARD.

Our findings must be interpreted with caution, as the varying designs of the trials and experience of the centers performing ECPR should be considered, as well as the uncertainty of the analyses, as reflected by the relatively wide credible intervals. Consequently, we believe that ECPR can be highly effective in ideal circumstances, which comprises dedicated treatment teams and a sufficient case load [40]. In such instances, the probability of clinically relevant results is highly likely. Instead, in less ideal circumstances, such as in inexperienced centers with rather low case volumes, ECPR may provide less benefit, and its implementation should perhaps be reconsidered. Clinical guidelines could aid in these processes by appointing centers of expertise and defining thresholds for case volumes.

Finally, we advocate for the use of a predefined Bayesian approach in randomized clinical trials studying diseases such as refractory OHCA and interventions like ECPR, given their relative infrequency, and the time- and cost-consuming nature of the intervention. It may indeed be implausible to achieve a sufficient sample size to detect a clinically relevant treatment effect under the frequentist statistical framework. For future trials, the Bayesian approach could facilitate the estimation of clinically relevant treatment effects, while the results of the current pooled analysis of RCTs can be used as an informed prior for such studies.

### Strengths and limitations

First, this meta-analysis only included RCTs, intended to reduce various forms of risk bias associated with non-randomized study designs. In addition, the study was performed by the authors of the three RCTs. Nevertheless, a potential limitation is related to the facts that the included RCTs had varying designs (single- or multi-center), varied markedly in sample size (i.e., ARREST comprised 30 patients), applied different randomization protocols, and Prague OHCA and INCEPTION were subjected to deviations from the intended treatment [4, 5].

Not all patients in the ECPR groups of the Prague OHCA and INCEPTION trials’ intention-to-treat analyses actually received ECPR [4, 5]. This could be a consequence of ECPR being part of a bundle of therapies (i.e. a hyperinvasive approach including ECPR in Prague OHCA [4]), the randomization process (i.e. pre-hospital randomization), the pre-hospital return of spontaneous circulation, or cross-overs. Although per-protocol data is available [43], the inclusion of such analyses exposes the current study to new forms of bias. Therefore, the term ‘ECPR-based treatment’ was used in this study.

The choice and specification of an informed prior within the Bayesian framework is heavily debated in the literature and may be exposed to criticism in terms of the relative subjectivity of its elicitation. Therefore, all analyses were conducted under a vague prior, assuming no difference between treatments with a wide distribution of possible treatment effects, ensuring optimal objectivity. In addition, there is wide variability in the determination of the MCID in ECPR research. We attempted to mitigate for this limitation by studying several other treatment effect sizes. Finally, some advocate for using fixed-effects models in meta-analyses with a relatively low number of trials. However, this artificially decreases the uncertainty of the observed effects. Therefore, we employed the appropriate random-effects model, which does not assume one true effect size to underlie all studies in the meta-analysis.

## Conclusion

The current Bayesian meta-analysis found a 71.1% and 75.8% probability of a clinically relevant ECPR-based treatment effect on 6-month neurologically favorable survival in refractory OHCA patients with all rhythms and shockable rhythms, respectively. The observed differences may be considered clinically relevant but must be interpreted within the context of the reported credible intervals and varying trial designs.

### Supplementary Information

Supplementary file1 (PDF 80 KB)

Supplementary file2 (DOCX 316 KB)

## Data Availability

All coding and analyses are openly shared and accessible through the corresponding author’s GitHub repository (https://github.com/samuelheuts/Bayesian_analysis_ECPRtrials) upon publication of this study.

## Abbreviations

ACR: Assumed control risk
ARD: Absolute risk difference
CCPR: Conventional cardiopulmonary resuscitation
CPC: Cerebral performance category
CI: Confidence interval
CrI: Credible interval
ECPR: Extracorporeal cardiopulmonary resuscitation
MCID: Minimal clinically important difference
MCMC: Markov Chain Monte Carlo
mRS: Modified Rankin Scale
NNT: Number needed to treat
OHCA: Out-of-hospital cardiac arrest
RCT: Randomized controlled trial
RoB: Risk of bias
RR: Relative risk
V-A ECMO: Veno-arterial extracorporeal membrane oxygenation
