# Supplementary material for: Extracorporeal cardiopulmonary resuscitation versus standard treatment for refractory out-of-hospital cardiac arrest: a Bayesian meta-analysis
Source: Crit Care. 2024 Jul 3;28:217. doi: 10.1186/s13054-024-05008-9 (PMC11223393; doi:10.1186/s13054-024-05008-9)
Supplement: Supplementary file 2 — Supplementary file2 (DOCX 316 KB) [file 13054_2024_5008_MOESM2_ESM.docx]

SUPPLEMENTARY MATERIAL

*Extracorporeal cardiopulmonary resuscitation versus standard treatment for refractory out-of-hospital cardiac arrest: A Bayesian meta-analysis*

Samuel Heuts, MD, PhD^1^, Hans Ubben, MD, Michal J. Kawczynski, MD, Andrea Gabrio, PhD, Martje M. Suverein, MD, Thijs S.R. Delnoij, MD, Petra Kavalkova, PhD, Daniel Rob, MD, PhD, Arnošt Komárek, PhD, Iwan C.C. van der Horst, MD, PhD, Jos G. Maessen, MD, PhD, Demetris Yannopoulos, MD, Jan Bělohlávek, MD, PhD, Roberto Lorusso, MD, PhD, Marcel van de Poll, MD, PhD

*Content Page*

Supplementary Material 1 – Detailed search 2

Supplementary Material 2 – Predefined worksheet 7

Supplementary Material 3 – Formulas for calculation 8

Supplementary Material 4 – Prior specification and rationale 10

Supplementary Material 5 – Risk of bias assessment 11

Supplementary Material 6 – Publication bias assessment 12

**Supplementary Material 1.** Detailed search for PubMed, EMBASE, and Cochrane Library.

| **Overview** | | |
| --- | --- | --- |
|  | | |
| Database | MEDLINE & PubMed Central | |
| Platform | PubMed | |
| Date of search | 21.11.2023 | |
| Number of results | 877 | |
| **Syntax guide** | | |
| [MeSH] | Medical subject headings | |
| Free search terms | Terms entered as “All fields” | |
| **Search** | **Query** | **Items found** |
| **Disease associated search terms** | | |
| #1 | Out-of-hospital cardiac arrest[MeSH] | 7.265 |
| #2 | Out-of-hospital cardiac arrest | 12.868 |
| #3 | OHCA | 4,440 |
| #4 | Ventricular fibrillation[MeSH] | 17,942 |
| #5 | Ventricular fibrillation | 46.941 |
| #6 | VF | 17.540 |
| #7 | #1 OR #2 OR #3 OR #4 OR #5 OR #6 | 69.665 |
| **Intervention associated search terms** | | |
| #8 | Extracorporeal cardiopulmonary resuscitation | 2.476 |
| #9 | ECPR | 865 |
| #10 | Extracorporeal CPR | 2.542 |
| #11 | Extracorporeal membrane oxygenation[MeSH] | 15.551 |
| #12 | Extracorporeal membrane oxygenation | 22.670 |
| #13 | ECMO | 24.224 |
| #14 | Veno arterial extracorporeal membrane oxygenation | 22.670 |
| #15 | Veno arterial ECMO | 1.971 |
| #16 | V-A ECMO | 255 |
| #17 | VA ECMO | 2.422 |
| #18 | #8 OR #9 OR #10 OR #11 OR #12 OR #13 OR #14 OR #15 OR #16 OR #17 | 24.921 |
| **Control treatment associated terms** | | |
| #19 | Cardiopulmonary resuscitation[MeSH] | 22.602 |
| #20 | Cardiopulmonary resuscitation | 33.123 |
| #21 | Conventional cardiopulmonary resuscitation | 1.020 |
| #22 | CPR | 39.960 |
| #23 | Advanced Life support | 78.099 |
| #24 | ALS | 58.319 |
| #25 | #19 OR #20 OR #21 OR #22 OR #23 OR #24 | 171.896 |
| **Combined search term** | | |
| #26 | #7 AND #18 AND #25 | 877 |

| **Overview** | | |
| --- | --- | --- |
| Database | Cochrane Library | |
| Platform | Cochrane Library | |
| Date of search | 21.11.2023 | |
| Number of results | 51 | |
| **Syntax guide** | | |
| [MeSH] | Medical subject headings | |
| Free search terms | Terms entered as “All fields” | |
| **Search** | **Query** | **Items found** |
| **Disease associated search terms** | | |
| #1 | Out-of-hospital cardiac arrest[MeSH] | 665 |
| #2 | Out-of-hospital cardiac arrest | 1.614 |
| #3 | OHCA | 618 |
| #4 | Ventricular fibrillation[MeSH] | 630 |
| #5 | Ventricular fibrillation | 4.424 |
| #6 | VF | 2.476 |
| #7 | #1 OR #2 OR #3 OR #4 OR #5 OR #6 | 7.777 |
| **Intervention associated search terms** | | |
| #8 | Extracorporeal cardiopulmonary resuscitation | 104 |
| #9 | ECPR | 99 |
| #10 | Extracorporeal CPR | 49 |
| #11 | Extracorporeal membrane oxygenation[MeSH] | 303 |
| #12 | Extracorporeal membrane oxygenation | 1.001 |
| #13 | ECMO | 1.010 |
| #14 | Veno arterial extracorporeal membrane oxygenation | 78 |
| #15 | Veno arterial ECMO | 95 |
| #16 | V-A ECMO | 7 |
| #17 | VA ECMO | 117 |
| #18 | #8 OR #9 OR #10 OR #11 OR #12 OR #13 OR #14 OR #15 OR #16 OR #17 | 1.495 |
| **Control treatment associated terms** | | |
| #19 | Cardiopulmonary resuscitation[MeSH] | 1.484 |
| #20 | Cardiopulmonary resuscitation | 3.021 |
| #21 | Conventional cardiopulmonary resuscitation | 229 |
| #22 | CPR | 2.848 |
| #23 | Advanced Life support | 4.143 |
| #24 | ALS | 3.263 |
| #25 | #19 OR #20 OR #21 OR #22 OR #23 OR #24 | 11.208 |
| **Combined search term** | | |
| #26 | #7 AND #18 AND #25 | 58 |
| #27 | Filter: clinical trials | 51 |

| **Overview** | | |
| --- | --- | --- |
| Database | Embase | |
| Platform | Embase library | |
| Date of search | 21.11.2023 | |
| Number of results | 1.153 | |
| **Syntax guide** | | |
| .af | Search term for all fields | |
| **Search** | **Query** | **Items found** |
| **Disease associated search terms** | | |
| #1 | Out-of-hospital cardiac arrest.af | 17.870 |
| #2 | OHCA.af | 8.201 |
| #3 | Ventricular fibrillation.af | 28.860 |
| #4 | VF.af | 39.026 |
| #5 | #1 OR #2 OR #3 OR #4 | 74.530 |
| **Intervention associated search terms** | | |
| #6 | Extracorporeal cardiopulmonary resuscitation.af | 1.586 |
| #7 | ECPR.af | 1.907 |
| #8 | Extracorporeal CPR.af | 151 |
| #9 | Extracorporeal membrane oxygenation.af | 27.294 |
| #10 | ECMO.af | 29.939 |
| #11 | Veno arterial extracorporeal membrane oxygenation.af | 2.039 |
| #12 | Veno arterial ECMO.af | 5.203 |
| #13 | V-A ECMO.af | 486 |
| #14 | VA ECMO.af | 4.486 |
| #15 | #6 OR #7 OR #8 OR #9 OR #10 OR #11 OR #12 OR #13 OR #14 | 39.493 |
| **Control treatment associated terms** | | |
| #16 | Cardiopulmonary resuscitation.af | 28.858 |
| #17 | Conventional cardiopulmonary resuscitation.af | 304 |
| #18 | CPR.af | 31.255 |
| #19 | Advanced Life support.af | 5.039 |
| #20 | ALS.af | 84.856 |
| #21 | #16 OR #17 OR #18 OR #19 OR #20 | 133.682 |
| **Combined search term** | | |
| #22 | #5 AND #15 AND #21 | 1.153 |

**Supplementary Material 2.** Predefined worksheet

| First author |
| --- |
| Study year |
| Country of study |
| Study design (single-center, multi-center) |
| Timing of randomization (pre-hospital, in-hospital) |
| OHCA type (shockable, non-shockable) |
| Number of patients (n) |
| Age (years, mean, SD) |
| Sex (female, n, %) |
| OHCA cause (AMI, other, n, %) |
| Bystander CPR (n, %) |
| Shockable rhythm (n, %) |
| Mechanical CPR (n,%) |
| Presenting lactate (mmol/L) |
| 30-day neurologically favorable survival (CPC 1-2, n, %) |
| 6-month neurologically favorable survival (CPC 1-2, n, %) |

*AMI: acute myocardial infarction, CPC: cerebral performance category, CPR: cardiopulmonary resuscitation, OHCA: out-of-hospital cardiac arrest, SD: standard deviation.*

**Supplementary Material 3.** Formulas to calculate RRs, 95% CI, log RR, SD, ARD, and NNT.

a= counts of those with event in ECPR

b= counts of those with no event in CCPR

c= counts of those with event in ECPR

d= counts of those with no event in CCPR

- *RR = (a/(a+b))/(c/(c+d))*
- *Log RR = ln (RR)*
- *Lower bound 95% CI = exp(ln(RR) – 1.96*sqrt(1/a+1/c-1/(a+b)-1/(c+d)))*
- *Upper bound = exp(ln(RR) + 1.96*sqrt(1/a+1/c-1/(a+b)-1/(c+d)))*
- *Standard deviation: (upper limit-mean)/1.96*
- $NNT= \frac{1}{ACR x (1-RR)}$ (ref^34^)*
- $ARD=100 x ACR x (1-RR)$(ref^34^)*
- $RR= \frac{OR}{1-p + (p x OR)}$ (ref)**

*ACR was derived from the weighted pooled 6-month survival prevalence in the control group (CCPR) ^34^.**In case the RR of the prior needed to be calculated from studies that only reported ORs, we applied the formulas as proposed by Grant et al.^47^*.
ACR: assumed control risk**, ARD: absolute risk difference, CCPR: conventional cardiopulmonary resuscitation, CI: confidence interval, ECPR: extracorporeal cardiopulmonary resuscitation, log: logarithmic function, NNT: number needed to treat, RR: relative risk, SD: standard deviation.***Supplementary Material 4.** Prior specification and rationale.

| *Priors* | Mean log RR | SD log RR | Rationale | Corresponding to RR | Corresponding to 95% CrI |
| --- | --- | --- | --- | --- | --- |
| **Primary analyses** | | | |  |  |
| Vague | 0 | 2 | Based entirely on the findings from the pooled data | 1.00 | 0.02 – 50.00 |
| **Secondary analysis (shockable OHCA)** | | | |  |  |
| Vague | 0 | 2 | Based entirely on the findings from the pooled data | 1.00 | 0.02 – 50.00 |

*CrI: credible interval, RR: relative risk, SD: standard deviation.*

**Supplementary Material 5.** Risk of bias assessment using the RoB 2.0 tool for randomized

trials.

**
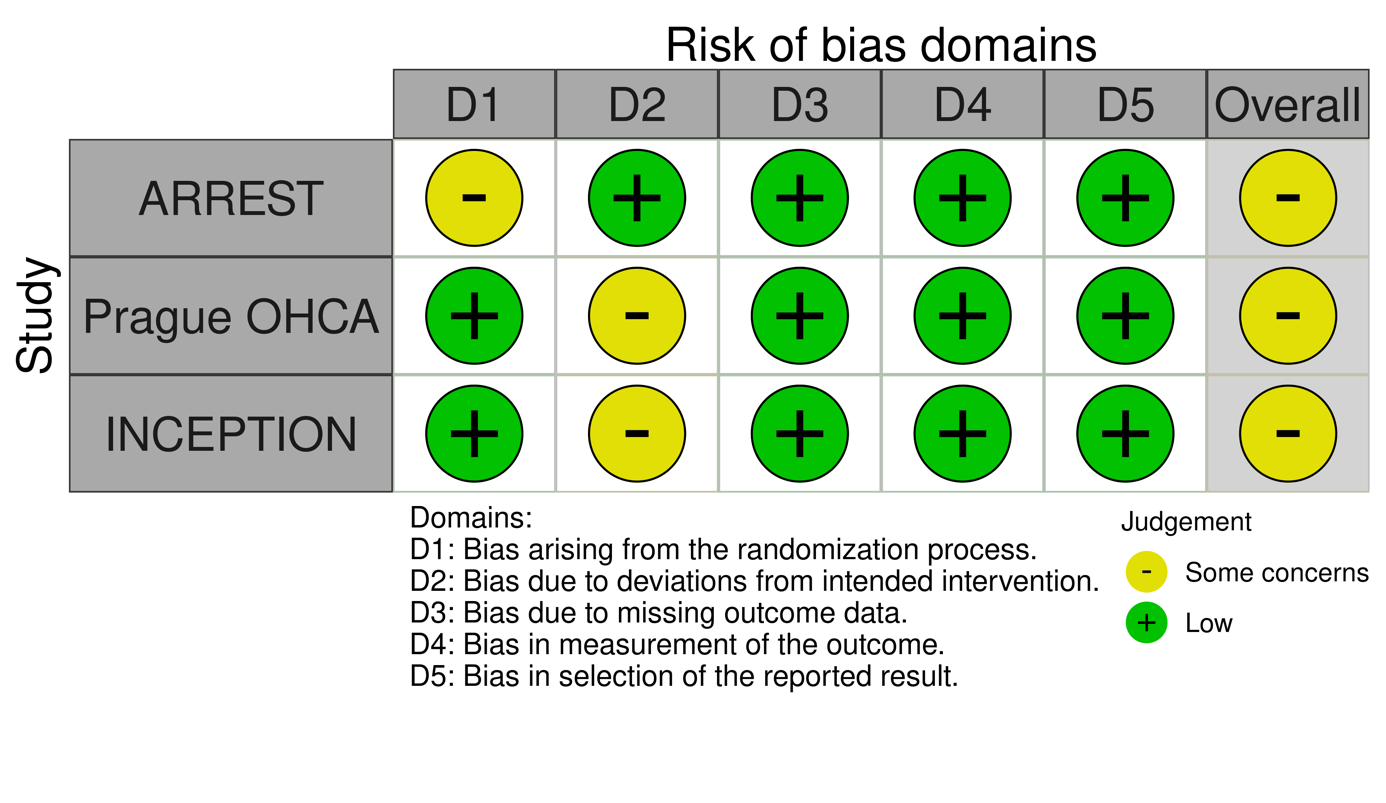
**

**Supplementary Material 6.** Funnel plot facilitating publication bias assessment with the result of the Egger’s test.


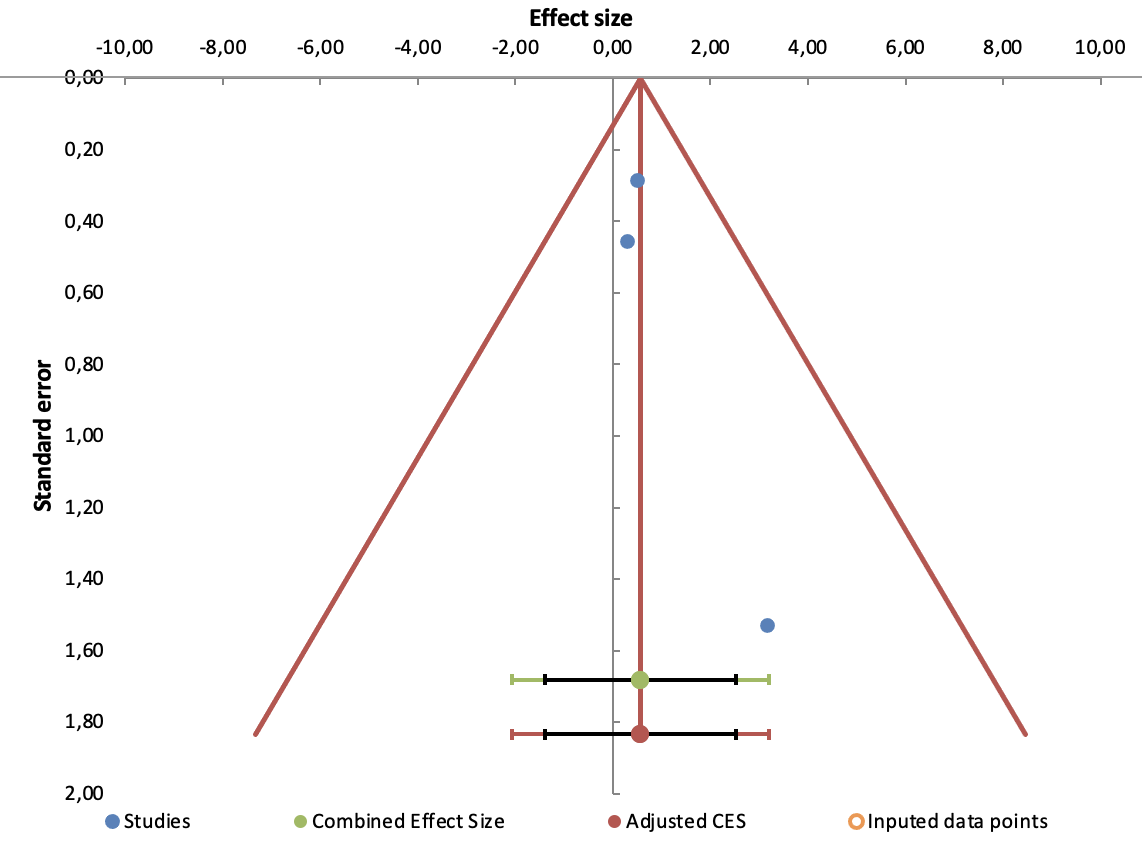


Egger’s test: p-value=0.239.

*CES: combined effect size.*
